# RBM10 C761Y mutation induced oncogenic ASPM isoforms and regulated β-catenin signaling in cholangiocarcinoma

**DOI:** 10.1186/s13046-024-03030-x

**Published:** 2024-04-04

**Authors:** Jiang Chang, Yaodong Zhang, Tao Zhou, Qian Qiao, Jijun Shan, Yananlan Chen, Wangjie Jiang, Yirui Wang, Shuochen Liu, Yuming Wang, Yue Yu, Changxian Li, Xiangcheng Li

**Affiliations:** 1 Hepatobiliary Surgery Hepatobiliary Center, The First Affiliated Hospital of Nanjing Medical University, 300 Guangzhou Road, Nanjing, Jiangsu Province China; 2 Key Laboratory for Liver Transplantation, Chinese Academy of Medical Sciences, NHC Key Laboratory of Living Donor liver Transplantation (Nanjing Medical University), Nanjing, Jiangsu Province China; 3 Wuxi People’s Hospital, Wuxi Medical Center, The Affiliated Wuxi People’s Hospital of Nanjing Medical University, Nanjing Medical University, Wuxi, China

**Keywords:** RBM10, Mutation, Alternative splicing, ASPM, Cholangiocarcinoma

## Abstract

**Background:**

Cholangiocarcinoma (CCA) comprises a heterogeneous group of biliary tract cancer. Our previous CCA mutation pattern study focused on genes in the post-transcription modification process, among which the alternative splicing factor RBM10 captured our attention. However, the roles of RBM10 wild type and mutations in CCA remain unclear.

**Methods:**

RBM10 mutation spectrum in CCA was clarified using our initial data and other CCA genomic datasets from domestic and international sources. Real-time PCR and tissue microarray were used to detect RBM10 clinical association. Function assays were conducted to investigate the effects of RBM10 wild type and mutations on CCA. RNA sequencing was to investigate the changes in alternative splicing events in the mutation group compared to the wild-type group. Minigene splicing reporter and interaction assays were performed to elucidate the mechanism of mutation influence on alternative splicing events.

**Results:**

RBM10 mutations were more common in Chinese CCA populations and exhibited more protein truncation variants. RBM10 exerted a tumor suppressive effect in CCA and correlated with favorable prognosis of CCA patients. The overexpression of wild-type RBM10 enhanced the ASPM exon18 exon skipping event interacting with SRSF2. The C761Y mutation in the C_2_H_2_-type zinc finger domain impaired its interaction with SRSF2, resulting in a loss-of-function mutation. Elevated ASPM203 stabilized DVL2 and enhanced β-catenin signaling, which promoted CCA progression.

**Conclusions:**

Our results showed that RBM10^C761Y^-modulated ASPM203 promoted CCA progression in a Wnt/β-catenin signaling-dependent manner. This study may enhance the understanding of the regulatory mechanisms that link mutation-altering splicing variants to CCA.

**Supplementary Information:**

The online version contains supplementary material available at 10.1186/s13046-024-03030-x.

## Introduction

Cholangiocarcinoma (CCA) is one of the most lethal cancers, which originates from epithelial cells lining the biliary tract or the liver tissue [1]. The global prevalence of CCA has risen in recent decades, with a notable surge in Asian countries [2]. The North East Thailand exhibit the highest age-standardized incidence rates (85 cases per 100,000) [3]. While radical resection presents promising curative prospects for individuals with early-stage CCA, postoperative outcomes reveal a median overall survival of 40 months, with a 5-year survival rate spanning from 25–40% [4]. Meanwhile, most patients are diagnosed at an advanced stage when not amenable to surgical resection. The advancement of next-generation sequencing facilitates a profound comprehension of CCA heterogeneous etiology, which has identified genomic alterations in different groups of CCA. Among them, IDH1, HER2, FGFR2, and BRAF mutations have played an important role in the targeted therapy of CCA [5].

Our previous study also identified several potential driver genes involved in key pathways, in which driver genes engaged in post-transcriptional modification processes captured our attention. We demonstrated a loss-of-function mutation of METTL14, a key RNA N6-adenosine methyltransferase writer [6]. Alternative splicing is also a crucial mechanism of post-transcriptional regulation, which generates functionally distinct splice variants at the RNA and protein levels [7]. Mutations in alternative splicing factors are also implicated in human cancers, especially hematological malignancies [8–10]. From the initial study, we noticed the mutation of RNA-binding protein 10 (RBM10) in the prevalence of CCA. RBM10 is an alternative splicing-related protein, whose mutation has been reported in lung adenocarcinoma (LUAD) (mutation rate 5–20%) [11, 12]. RBM10 is recognized as a tumor suppressor whose mutations significantly alter mRNA isoform expression, thereby compromising its anti-tumor function [13]. Unlike the hotspot mutations of oncogenes, the mutations of tumor suppressor genes occur throughout the gene, leading to different effects on essential structures. We detected a missense mutation (C761Y) in the C_2_H_2_-type zinc finger domain of RBM10, a highly conserved and crucial domain for its role in regulating alternative splicing [14, 15]. The function of wild-type RBM10 in CCA and the effect of its mutation in this structure have never been elucidated before. In this study, we explored the role of RBM10 in the progression of CCA and the impact of this mutation. These results provide new insights into the involvement of mutation-altering alternative splicing in cholangiocarcinoma.

## Materials and methods

### RNA sequencing and Sanger sequencing

Total RNA was extracted from QBC939 cells in the negative control (NC), wild-type (WT), and mutant (MUT) groups using TRIzol reagent (Invitrogen, California, USA). RNA sequencing and analysis were performed by LC Sciences (Hangzhou, China) using an Illumina HiSeq2500 platform. Alternative splicing (AS) events were quantified by rMATS version 4.1.0 [16]. AS events with significant changes between WT and MUT were identified based on the criteria of false discovery rate (FDR) < 0.01 and | inclusion level difference| > 0.1, as calculated by rMATS. Matched Annotation from NCBI and EMBL-EBI (MANE) transcript was considered as the representative transcript per gene according to the Ensembl database in ES event analysis, which means a poorly expressed transcript was excluded regardless of the abundant expression of other transcripts.

Sanger sequencing was performed by Sangon Biotech (Nanjing, China) to confirm the presence of the RBM10 C761 mutation in cultured cells. The forward primer of RBM10 in this study is 5’-TCTGATCTGGCCAGGCCTGA-3’ and the reverse primer is 3’-CTCGCCGGTGAATCTCAAGG-5’.

### Evaluation of immunostaining

Hematoxylin and eosin (H&E) staining was performed routinely. Tissue microarrays containing 177 cases of CCA specimens and corresponding adjacent normal tissues, were obtained from The Affiliated Hospital of Nanjing Medical University. Scoring was conducted as described in our previous study [6]. The study cohort underwent regular follow-up until either mortality or October 25, 2019. The Ethics Committee of The Affiliated Hospital of Nanjing Medical University approved the utilization of clinical specimens.

### Cell cultures and transfection

Human CCA cell lines (QBC939, RBE) were obtained from the Cell Bank of Chinese Academy of Sciences (Shanghai, China) and cultured in Dulbecco’s Modified Eagle Medium (Gibco, CA, USA) supplemented with 10% FBS (FBS; Gibco) and 100 units/ml of penicillin/streptomycin (Gibco). All cells were maintained at 37°C in a humidified atmosphere of 5% CO2. The plasmid containing pcDNA-DVL2 and siRNA against ASPM exon18 were purchased from Gene Pharma (Shanghai, China). Sequence for the siRNA of ASPM203: 5’-GAUAUAGAGCCAAGAAAUATT-3’. Sequence for siRNA of SRSF2: 5’-GUGAGAAGUUGCUUAGAAATT-3’.

### Establishment of stable RBM10^C761Y^ and RBM10^WT^ cells and function assay

Recombinant lentiviruses were produced by using vectors that encoded empty control, RBM10^C761Y^, and RBM10^WT^ gene. These vectors were designed by GeneChem (Shanghai, China). The lentivirus transfection assay was conducted on 2 × 10^5^ cells following the manufacturer’s protocol. To select stable cells, puromycin (10 µg/ml) was administered for 7 days. Cell counting kit (CCK)-8 assay, colony formation assay, 5-ethynyl-20-deoxyuridine (EdU) incorporation assay, transwell assay, and wound-healing assay were performed as described in our previous study [6].

### RNA extraction and quantitative real-time PCR and RNA immunoprecipitation (RIP)

Total RNA from cell lines or tissues was extracted using the RNA-Quick Purification Kit (Yishan, Shanghai, China) following the manufacturer’s instructions. The RNA purity and concentration were measured by NanoDrop ND2000 (Thermo, USA). We synthesized cDNA using Primescript RT Reagent (Vazyme, China). Real-time PCR was conducted with a standard SYBR Green PCR kit (Vazyme, China) using the Thermal Cycler Dice Detection System (ABI 7900; Thermo, USA). The relative expression of target genes was calculated using GAPDH as the reference gene. The primers used for this study were listed in Table S3 . RIP was done using the Geneseed RIP kit (Geneseed, Guangzhou, China) according to the protocol provided. Antibodies used were anti-RBM10 (Novus, NB100-55265), anti-SRSF2 (Santa Cruz, SC-13,509), and IgG (Cell Signaling Technology, Cat No. 2729).

### Western blotting and immunofluorescence assay

Protein extraction from cells and tissue samples was performed using the RIPA reagent kit (Beyotime, Shanghai, China). Proteins were separated by SDS-PAGE (6-12.5% sodium dodecyl sulfate-polyacrylamide gel electrophoresis) and transferred to polyvinylidene difluoride membranes (Millipore, USA). The membranes were blocked with QuickBlock™ Blocking Buffer for Western Blot (Beyotime, Shanghai, China) for 15 min and incubated with primary antibodies overnight at 4℃, followed by secondary antibodies for 2 h at room temperature. Protein detection was carried out with an ultra-sensitive ECL chemiluminescent substrate (Bioshrap, Anhui, China). Immunofluorescence assay performed as described in our previous study [6].The antibodies used in this study were listed in Table S3.

### Minigene splicing reporter assays

To study ASPM exon18 splicing following RBM10^WT^ or RBM10C^761Y^ overexpression, QBC939 and RBE cells were transfected with minigene splicing reporters. The minigene splicing reporters for test exons (ASPM exon 17, truncated exon18 (498 bp), exon 19, and exon18 flanking introns) was constructed by PharmaCore Labs (Jiangsu, China). The ASPM minigene-specific sequences were listed in Table S3. To analyze the RNA, the cells were collected 48 h post-transfection. The splicing patterns were quantified by calculating the percent spliced-in (PSI) values.

### RNA pull-down assay and mass spectrometry (MS) analysis

RNA pull-down assay was done using the Pierce™ Magnetic RNA-Protein Pull-Down Kit (Thermo, USA) following the manufacturer’s instructions. In the pull-down assay, we employed a plasmid containing the ASPM mini gene (comprising only exons truncated 17–19 and flanking introns). Positive and negative probes were constructed by RiboBio company (Shanghai, China). The pull-down protein bands were detected by silver staining and analyzed by MS (BGI Genomics, Shenzhen, China).

### Immunoprecipitation (IP)

For the immunoprecipitation assay, antibodies were incubated with BeyoMag™ Protein A + G magnetic beads (Beyotime) overnight at 4℃. The beads were then washed with elution buffer to remove the excess antibodies. The antibody-bound magnetic beads were mixed with the protein lysate and agitated at room temperature for 120 min. The immunoprecipitated proteins were eluted with elution buffer and separated by SDS-PAGE.

### Animal experiments

The animal experiments were conducted following the protocols approved by the Institutional Animal Care and Use Committee and the Ethical Committee of Nanjing Medical University. Male BALB/c nude mice (4 weeks old) were obtained from the Animal Core Facility of Nanjing Medical University (Nanjing, China). QBC939 cells transfected with lentivirus expressing RBM10^NC^, RBM10^WT^, and RBM10^C761Y^ (5 × 10^6^ cells in 100 µL of serum-free PBS) were injected subcutaneously into the flanks of the mice. The mice were sacrificed after four weeks. Tumor volume was calculated using the formula: volume = length × width^2^ × 0.5. The maximum tumor diameter was limited to 1.5 cm.

### Datasets

Data of our cohort (67 CCAs ) was obtained from our previous study [6]. Data of China pan-cancer cohort, TCGA cohort, and MSK-IMPACT Clinical Sequencing Cohort was obtained from Cbioportal (https://www.cbioportal.org/).

### Statistical analysis

Statistical tests for all experimental results were carried out using GraphPad Prism 8 (GraphPad Software, La Jolla, USA) and SPSS v26.0 (IBM, Chicago, USA). Two-tailed Student’s t-tests were used for comparisons between sample pairs. Survival without disease recurrence (disease-free survival, DFS) and overall survival (OS) were estimated by the Kaplan‒Meier method and compared by the log-rank test. A Cox proportional hazard regression model was fitted to identify independent prognostic factors. Pearson rank correlation analysis was performed to evaluate the association between RBM10 expression and ASPM203/201 relative expression. Multiple testing corrections were performed where necessary using the Benjamini-Hochberg method. *P < 0.05, **P < 0.01 and ***P < 0.001 are used for all the analyses.

## Results

### RBM10 mutations frequently occurred in CCA

**Fig. 1:**
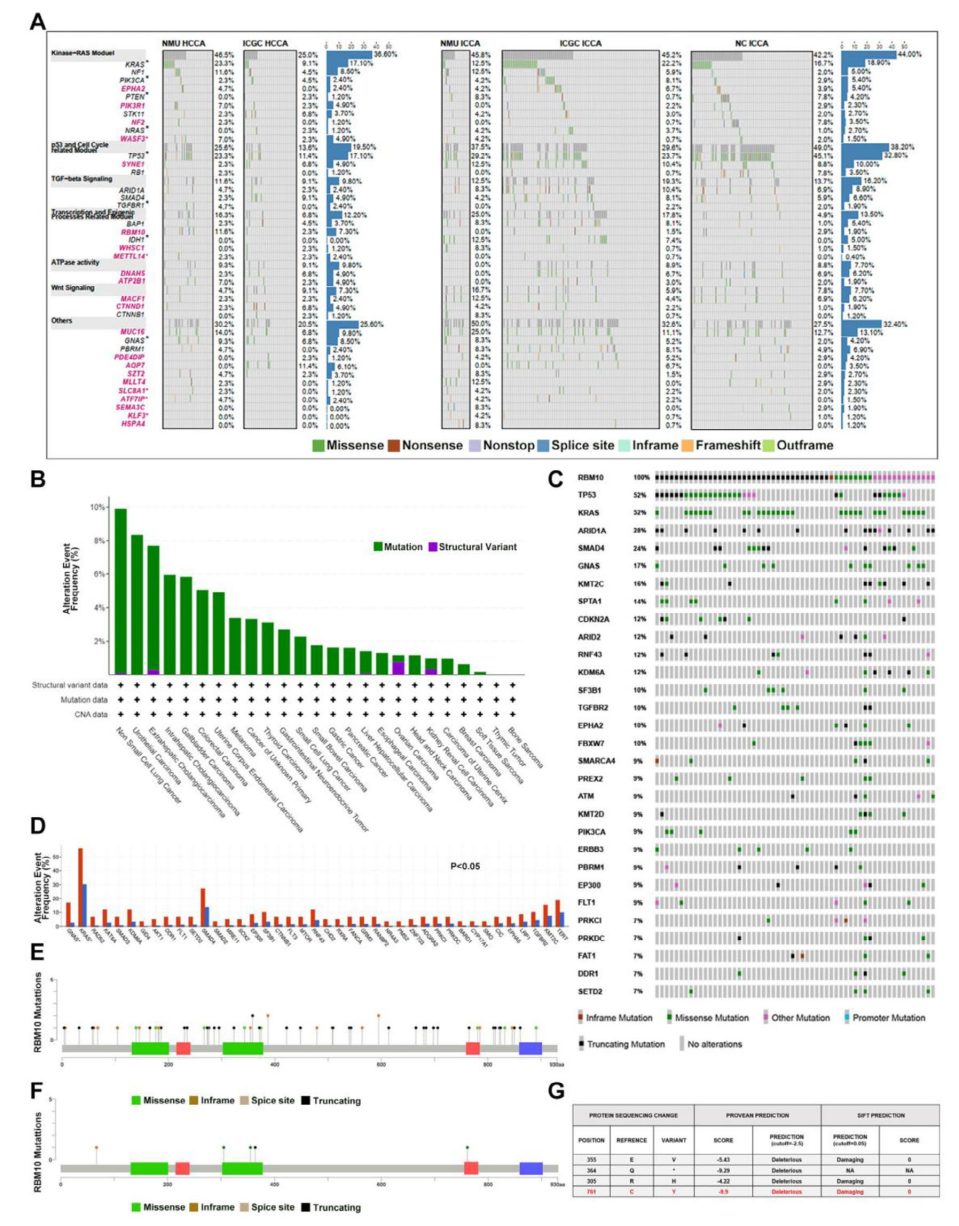
Mutation spectrum of RBM10 in CCA. **A** Functional cluster analysis was conducted in our previously identified potential driver genes in CCA. The gene symbols in red represented the CCA-related genes that were identified in our previous study. Gene symbols in black were previously reported in CCA. NMU, our own CCA cohort (67 cases); ICGC, International Cancer Genome Consortium (179 cases); NC Zou et al.’s study [38] (102 cases). **B** RBM10 alteration frequency in pan-cancer in Chinese population from Cibioportal. **C** Top 30 Concurrent gene alterations in CCA patients with RBM10 mutations in the Chinese population. **D** Significant alteration rate difference between RBM10 alter and unalter groups in CCA (*P* < 0.05). Blue column RBM10 unalter group; red column, RBM10 alter group. **E** RBM10 point mutation distribution in 58 of 859 CCA samples in the Chinese population. Lollipop plot showed mutations throughout the RBM10 gene area, using MutationMapper. Green rectangle, two RNA recognition motifs (RRM); red rectangle, two zinc fingers (left, C4-type zinc finger domain; Right, C2H2-type zinc finger domain); purple rectangle, Octamer repeat domain(OCRE). **F** RBM10 point mutation distribution in 5 of 67 CCA samples from our dataset. **G** Pathogenicity prediction of mutations in our cohort using PROVEAN and SIFT

In the previous integration analysis of significantly mutated genes, we highlighted the effect of the post-transcriptional modification gene set on CCA development (Fig. 1. A). Notably, in our cohort, approximately 7.5% (5/67) of CCA patients harbored RBM10 mutations, an alternative splicing factor involved in RNA maturation. The same result was validated in a large-scale pan-cancer Chinese population by exome sequencing [17]. The mutational landscape of RBM10 in solid tumors revealed high mutation rates in both extrahepatic cholangiocarcinoma (7.4%, 26/351) and intrahepatic cholangiocarcinoma (6.3%, 32/508) in the Chinese populations(Fig. 1. B), whereas Western populations exhibited lower frequencies (0%, 0/36 in the TCGA population; 0.5%, 1/200 in the MSK-IMPACT population [18]) (Fig S1. A & B), hinting at a potential specificity of RBM10 mutations to Chinese CCA patients. Moreover, the RBM10-alter group showed a higher mutation count (*p* = 7.99e-7) and tumor mutation burden (*p* = 2.79e-7) than the unaltered group (Fig S1. D & E). We further analyzed the mutation patterns in the RBM10 alter group in Chinese CCA population cohort: classic tumor driver genes (KRAS, SMAD4, CTNNB1, TGFBR2, SF3B1, etc. *P* < 0.05) were more prone to co-occur in the RBM10 alter group (Fig. 1. C-D); the majority of RBM10 mutations were protein-truncating variants, including splice site, nonsense, and frameshift mutations (Fig. 1. E). Owing to the same high mutation rate of RBM10 in LUAD, RBM10 has been investigated and is suggested to play an important role in LUAD [19]. The RBM10 mutation pattern in LUAD mirrored those in CCA(Fig S1. C), resulting in RBM10 expression decreasing (Fig S1. F). RBM10 mutations also comprised a substantial proportion of missense mutations in CCA and LUAD, whose functional impacts were mostly ambiguous and needed experimental validation. Within our initial data, we detected three missense mutations, one nonsense mutation, and one splice site mutation. The three missense mutations affected two crucial domains (R305H and E355V in the second RNA recognition motif; C761Y in the C_2_H_2_-type Zinc finger domain) (Fig. 1. F). The effects of mutations in the second RNA recognition motif have been reported [11, 20], whereas the consequences of alterations in the C_2_H_2_-type zinc finger domain remain largely unexplored. Consequently, the in-silico pathogenicity prediction also indicated that RBM10^C761Y^ occurring in the C_2_H_2_-type zinc finger domain by PROVEAN and SIFT respectively, deserved further investigation (Fig. 1. G).

### RBM10 wild-type was downregulated in CCA and indicated worse survival

**Fig. 2:**
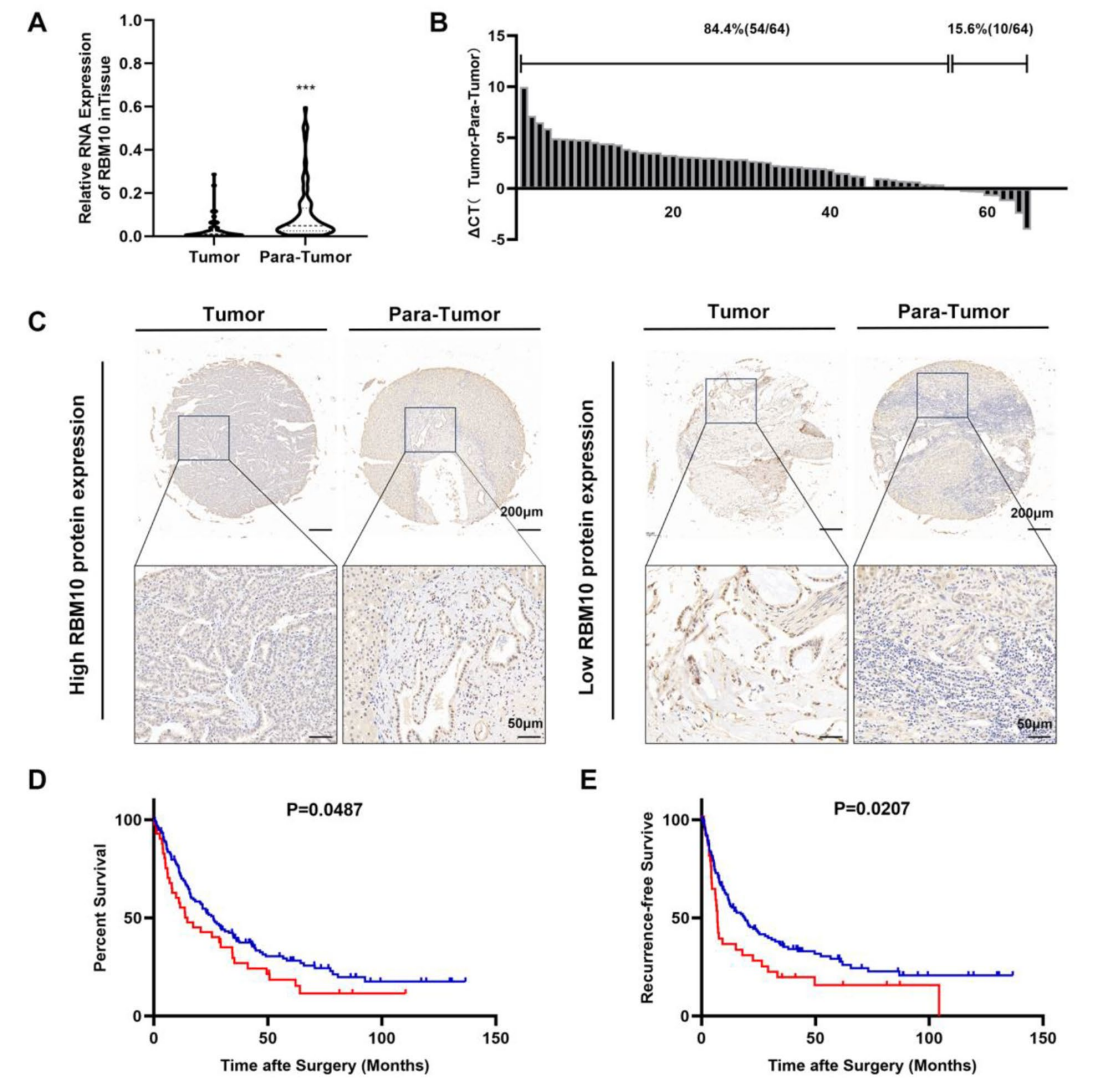
RBM10 was downregulated in CCA. **A** The different levels of RBM10 mRNA expression in paired CCA and adjacent tissues (*n* = 64). **B** The individual RBM10 mRNA expression difference distribution between tumor and para-tumor. **C** Representative IHC stains of RBM10 in CCA and adjacent normal tissues. **D, E** The prognostic value of RBM10 expression in CCA was evaluated by Kaplan-Meier analysis of overall survival (OS) and recurrence-free survival (RFS) according to the IHC staining intensity of RBM10 in TMA (*n* = 177). Blue line, high RBM10 expression in CCA; red line, low RBM10 expression in CCA

To elucidate the role of RBM10 mutations in CCA development, the clinical associations of wild-type RBM10 expression were initially examined in CCA. Analysis of RBM10 mRNA expression in 64 CCA patients revealed elevated levels in adjacent normal tissues compared to CCA tissues (Fig. 2. A & B). We subsequently assessed the protein expression of RBM10 in CCA tissue microarrays by immunohistochemistry (IHC) and observed the same trend. The representative images of RBM10 expression in tumor and para-tumor tissues were displayed (Fig. 2. C). Follow-up data based on CCA tissue microarrays confirmed RBM10 downregulation in tumors correlating with reduced overall survival (*P* = 0.0487) and recurrence-free survival (*P* = 0.0207) (Fig. 2. D & E), suggesting RBM10’s potential as a tumor suppressor gene in CCA. Furthermore, the multivariate analysis showed that RBM10 wild-type expression was an independent predictive factor for postoperative overall survival (*P* = 0.039) (Fig S1. G).

### RBM10^C761Y^ was identified as a loss-of-function mutation in CCA

**Fig. 3:**
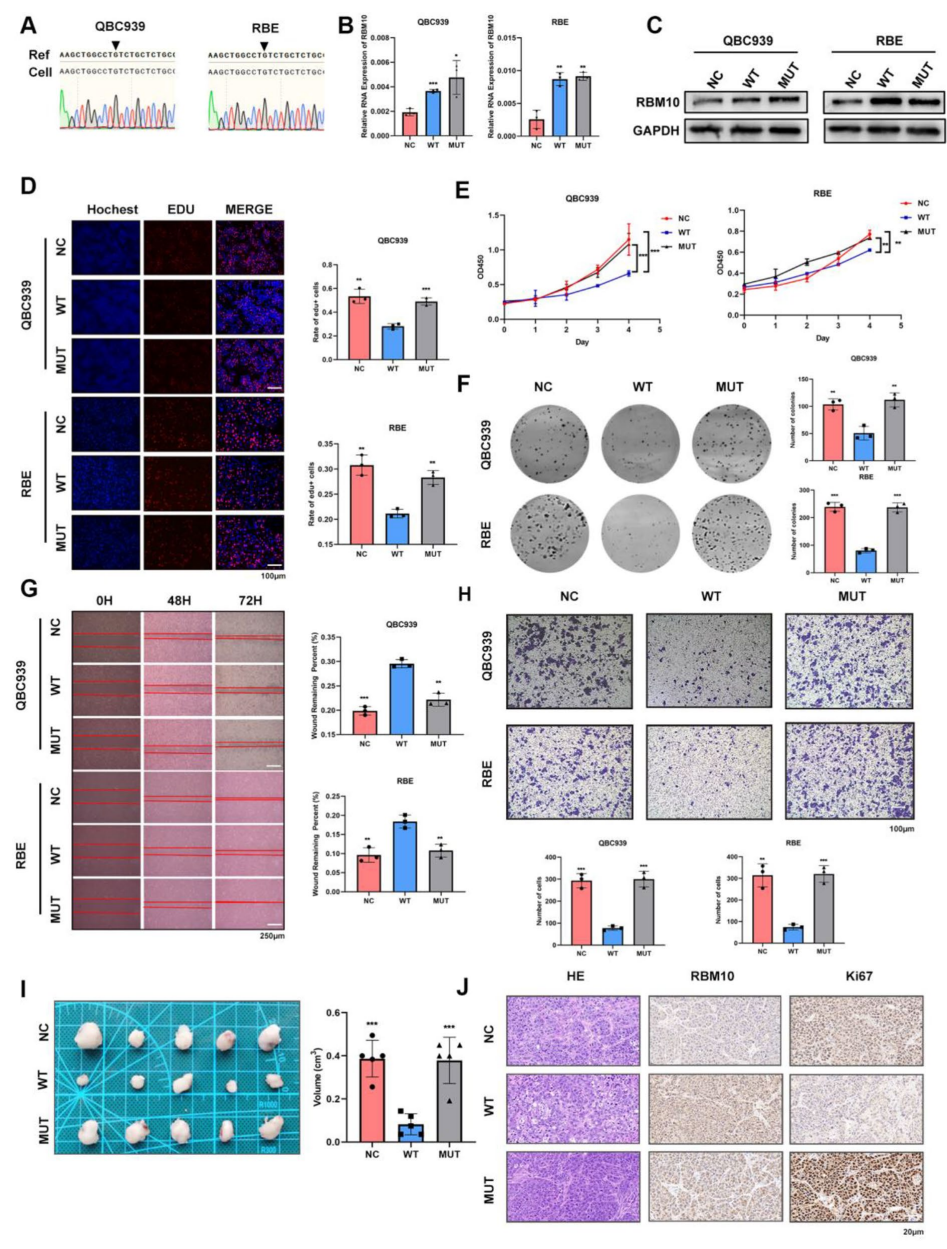
RBM10 was a tumor suppressor and RBM10^C761Y^ was a loss-of-function mutation in CCA. **A** Sanger sequencing plot of interested region including RBM10 C761Y in QBC939 and RBE. **B, C** Establishment of stable WT and MUT cells was evaluated at mRNA and protein levels. NC, negative control; WT, wild-type; MUT, mutant. **D, E, F** Proliferation ability of different groups was examined by EdU, CCK-8, and colony formation assay. **G, H** Migration ability of different groups was detected by wound-healing and transwell assay. **I** Photograph of excised tumors from mice in different groups (*n* = 5). **J** Representative images showed H&E and IHC staining for RBM10 and Ki67 in tumors removed from nude mice

To mitigate the influence of background RBM10, we assessed the expression levels of RBM10 in four CCA cell lines (QBC939, RBE, HuCCT1, and HCCC9810) and one normal bile duct cell line (HiBEC) before establishing experimental cell models. We selected two cell lines, QBC939 and RBE, which exhibited the lower expression levels of RBM10 (Fig S2. A & B). Then, we establish two stable RBM10^WT^/ RBM10^C761Y^ overexpressing CCA cell lines. The Sanger sequencing was performed to confirm the absence of RBM10^C761Y^ mutations in wild-type CCA cell lines (RBE and QBC939) (Fig. 3. A) and the transduction efficiency of lentiviral vectors was evaluated at RNA and protein levels (Fig. 3. B & C). Additionally, the C761Y mutation was detected exclusively in the MUT group by designed mutation-specific primers (Fig S2. C & D). We observed fewer EdU-positive cells in the wild-type (WT) group than in the negative control (NC) group. However, the mutant (MUT) group significantly restored this ability (Fig. 3. D). The cell viability of CCA cells was assessed by Cell Counting Kit-8 (CCK-8) assay, which showed that the WT group exhibited lower cell viability than the NC group, while the MUT group showed no significant difference from the NC group (Fig. 3. E). Colony formation assay demonstrated the consistent alteration of proliferation ability (Fig. 3. F). In addition, overexpression of wild-type RBM10 inhibited CCA cell migration. By contrast, RBM10^C761Y^ lost the tumor-suppressive functions, as shown by the transwell and wound healing assay (Fig. 3. G&H).

To elucidate the in vivo functions of RBM10^WT^ and RBM10^C761Y^ in CCA development, these CCA cells were inoculated subcutaneously into nude mice. Xenografts derived from QBC939 cells with stable wild-type RBM10 overexpression were smaller than those from control QBC939 cells. In agreement with the in vitro findings, the suppressive effects of RBM10^C761Y^ on tumor growth were significantly reduced in vivo compared with those of RBM10^WT^ (Fig. 3. I). The expression of Ki67 was decreased in the WT group, while no significant change was observed in the MUT group compared with the NC group (Fig. 3. J). Combined, these data suggested that the loss-of-function mutation RBM10^C761Y^ impaired the tumor suppressive role of wild-type RBM10 in CCA.

### ASPM alternative splicing served as the target of RBM10

**Fig. 4:**
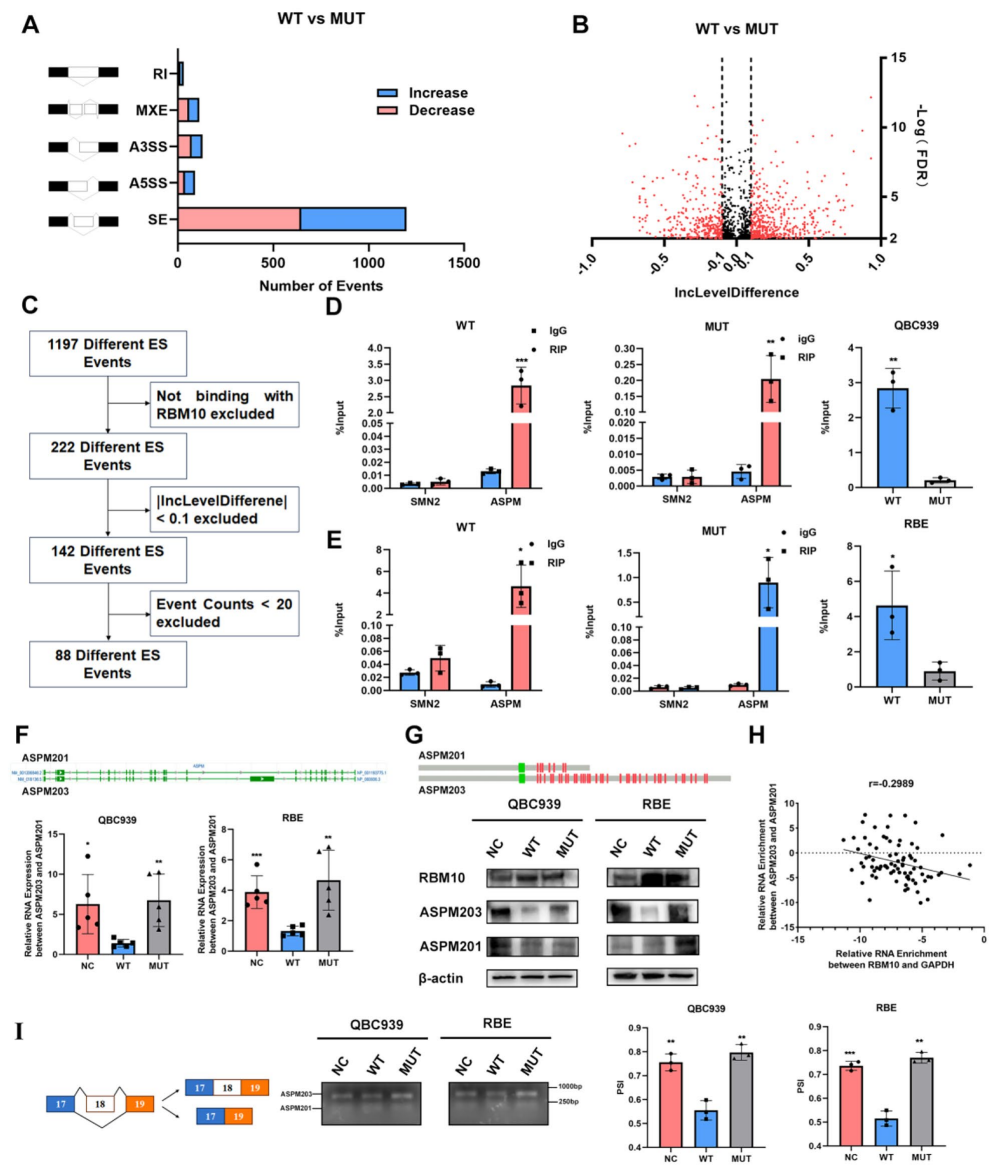
RBM10 modulated alternative splicing of ASPM in CCA. **A** The number of different alternative splicing events associated with RBM10^C761Y^ mutation in CCA. RI, retained intron; MXE, mutually exclusive exon; A3SS, alternative 3’ splice site; A5SS alternative 5’ splice site; SE, skipped exon. **B** Significant exon skipping (ES) changes between RBM10 WT and MUT groups (FDR < 0.01, |inclusion level differences| > 0.1). **C** A flowchart of exploring RBM10 mutation-related ES events. **D, E** RNA immunoprecipitation using anti-RBM10 or anti-SMN2 antibodies, or igG in different groups. RNA immunoprecipitation (RIP) products were analyzed by RT-PCR. The recovered RNA in each RIP was normalized to its input. **F, G** Isoforms expression of ASPM in NC, WT, and MUT groups were evaluated at mRNA and protein levels. Green rectangle in (**G)**, CAMSAP CH domain; red rectangle in (**G**), isoleucine and glutamine domain. **H** RT-PCR results revealed that the relative expression of ASPM203/ASPM201 was negatively related to RBM10 expression in CCA samples(*n* = 77) by Pearson rank correlation analysis (*r*=-0.2989, *P* = 0.0074). **I** The effects of RBM10 WT and MUT on ASPM exon18 ES event were examined using minigene splicing reporter assays

RBM10 modulated the alternative splicing of thousands of target genes and may influence the progression of CCA by altering the splicing patterns of cancer-related genes. To gain a deeper insight into the mechanism by which RBM10^WT^ and RBM10^C761Y^ functioned in CCA development, we performed RNA sequencing in the NC, WT, and MUT groups. Splicing changes caused by RBM10^C761Y^ in CCA cell lines were analyzed using the rMATS pipeline, which identified five types of alternative splicing events: retained intron, mutually exclusive exons, alternative 3’ splice site, alternative 5’ splice site, and skipped exon. The most obvious changes were exon skipping (ES) events, which were also the most prevalent events involving RBM10 (Fig. 4. A). Compared to the WT group, 1197 significant ES events (false discovery rate (FDR) < 0.01) were defined as RBM10^C761Y^-associated ES events (Fig. 4. B). Based on RBM10 cross-linking immunoprecipitation (CLIP) sequencing data [21, 22], inclusion level difference between the two groups and ES events count, we excluded 975, 80, and 54 ES events respectively. The remaining 88 ES events were defined as RBM10^C761Y^-modulated ES events. The 18th exon skipping of assembly factor for spindle microtubules (ASPM) was among the top significant ES events (Fig. 4. C & Table S1). To minimize the confounding effects of different cell lines on CLIP sequencing, we also identified SMN2 exon6 event as potential targets of RBM10^C761Y^ regulation by integrating the rMATS analysis results and the changes of the predominant transcripts in the sequencing results, regardless of the CLIP results (Fig S3. A & Table S2). In both the WT and MUT groups, we employed RNA immunoprecipitation experiments to determine which gene specifically binds to RBM10. The experimental results revealed that ASPM interacts with both the wild-type RBM10 and the C761Y mutant, however, the binding capacity of the C761Y mutant to ASPM was diminished (Fig. 4. D & E). These results implied that RBM10 may be involved in the ASPM ES event and C761Y mutant impaired the interaction between ASPM and RBM10.

The ASPM exon18 ES event resulted in two different transcripts (ASPM203 and ASPM201). The changes of ASPM203/ASPM201 were evaluated at RNA and protein levels in three groups of cells. The expression of ASPM203 in the WT group was lower than that in the NC group, while the MUT group did not show significant changes compared to the NC group (Fig. 4. F & G). Further analysis of RNA from CCA tissues revealed that the ratio of ASPM203 to ASPM201 was negatively correlated with the expression of RBM10 (Fig. 4. H *P* = 0.0074, *r*=-0.2989). We also performed ASPM minigene splicing reporter assays to explore the effects of the mutations on ASPM ES event. Compared with the NC group, the WT group showed significant changes in the splicing of ASPM minigene, while the MUT group did not affect the splicing (Fig. 4. I). Together, these observations demonstrate the ASPM exon18 ES events may serve as the target of RBM10.

### RBM10^C761Y^ reduced interaction with SRSF2 and influenced the ASPM ES event

**Fig. 5:**
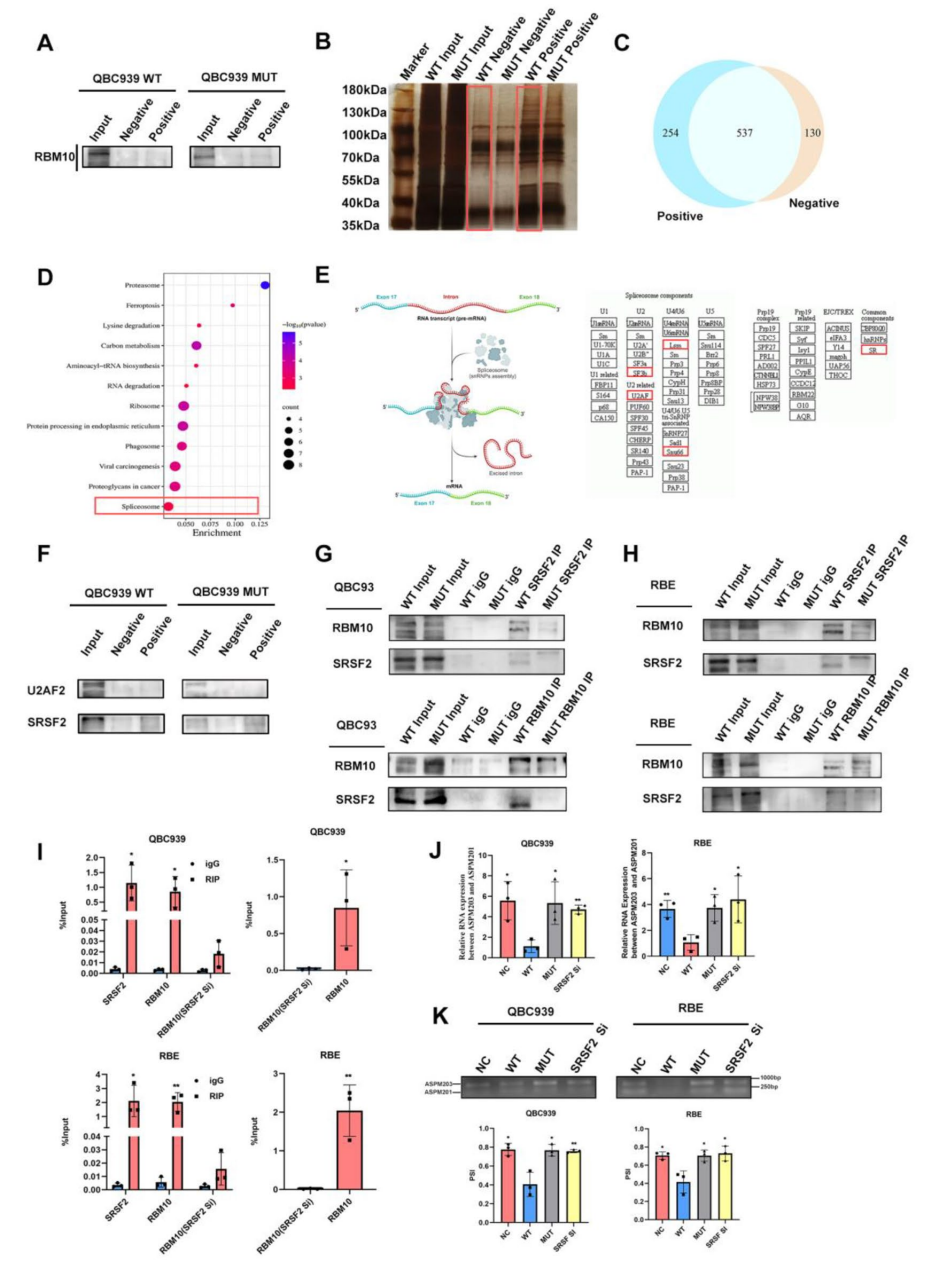
RBM10 and SRSF2 cooperatively controlled the splicing of ASPM. **A** Immunoblot analyses of RBM10 in ASPM-pulldowns derived from the WT and MUT groups transfected with ASPM-minigene constructs. **B** Sliver staining of ASPM-pulldowns. MS analysis was performed in WT groups. **C** Venn diagram of ASPM-pulldowns between positive and negative probes. **D** Pathway enrichment analysis of positive probe unique ASPM-pulldowns. **E** Potential spliceosome factors involved in ASPM ES. **F** Immunoblot analyses of U2AF2 and SRSF2 in ASPM-pulldowns. **G, H** Co-immunoprecipitation was performed to confirm the interaction between SRSF2 and RBM10/RBM10^C761Y^. RBM10 mutation weakened the interaction between SRSF2 and RBM10. **I** RNA immunoprecipitation using anti-RBM10 or anti-SRSF2 antibodies, or igG with SRSF2 knockdown. **J** RT-PCR was performed to detect the expression of ASPM isoforms in NC, WT, MUT, and SRSF2-knockdown groups. **K** Effects of RBM10 and SRSF2 on ASPM exon18 ES event were examined using minigene splicing reporter assays

To further understand how RBM10^C761Y^ mutation affected the ASPM ES event, the ASPM pull-down assay was conducted to explore the proteins involved in this ES event. However, RBM10 was not detected in the isolated pull-down products (Fig. 5. A). To identify more alternative splicing factors in the event, we further conducted silver staining and mass spectrometry (MS) analysis on the pull-down products (Fig. 5. B). There were 254 binding proteins in the positive probe group (Fig. 5. C). We focused on spliceosome-related proteins and found U2AF2 and SRSF2 as two potential candidates, which played important roles in alternative splicing [23, 24] (Fig. 5. D & E). RNA pull-down assay indicated that SRSF2 bound to ASPM minigene (Fig. 5. F). Co-immunoprecipitation experiments revealed that SRSF2 could form a complex with RBM10 wild type, while the mutant binding ability decreased in two cell lines (Fig. 5. G & H). Further RNA immunoprecipitation experiments showed that SRSF2 can bind to ASPM, and knocking down SRSF2 significantly reduced the binding ability of RBM10 to ASPM (Fig. 5. I). SRSF2 knockdown increased the ratio of ASPM203 to ASPM201 in the WT group at the RNA level (Fig. 5. J). The ASPM minigene splicing reporter assays also revealed that knocking down SRSF2 impaired the ability of the RBM10 wild type to regulate the splicing event of ASPM minigene (Fig. 5. K). The above results suggested that RBM10 interacted with SRSF2 and functioned in the ASPM exon18 ES event, while RBM10^C761Y^ reduced the connection.

### RBM10^C761Y^-modulated ASPM203 involved in CCA development

**Fig. 6:**
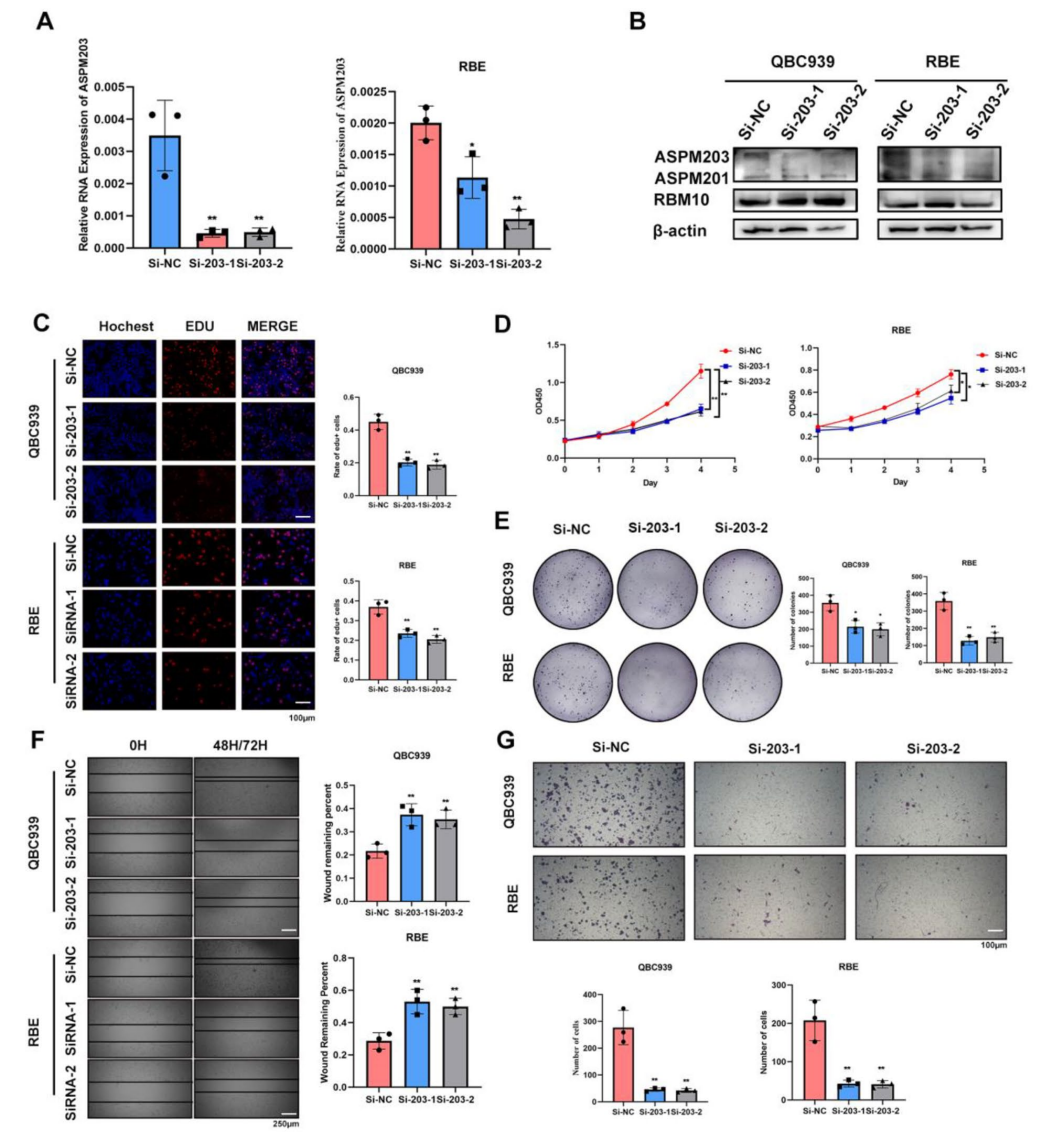
RBM10^C761Y^-mediated oncogenic ASPM isoforms involved in CCA development. **A, B** ASPM isoform expressions were examined in the MUT group with specific ASPM203 knockdown at mRNA and protein levels. **C** Edu assay, **D** growth curve, **E** and colony formation assay were performed to assess the effects on the proliferation capacity of CCA cell lines after knocking down ASPM203 in the MUT group. **F** Wound healing assay and **G** transwell assay were performed to assess the effects on the migration capacity of CCA cell lines after knocking down ASPM203 in the MUT group. Si-203: Si-ASPM203

To verify the role of ASPM203 in RBM10^C761Y^-mediated CCA progression, we silenced ASPM203 in the NC and MUT groups using siRNA. Silencing ASPM203 in the NC group reduced the proliferation and migration of CCA cell lines (Fig S4. A & B). We also found that ASPM mutations in TCGA CCA populations were all amplification mutations (Fig S4. C), suggesting that ASPM may facilitate the development of CCA. The mean percent spliced-in index of ASPM203 in pan-cancer was above 0.8, indicating that ASPM203 might be involved in cancer progression (Fig S4. D). The use of RNAi targeting exon18 sequences did not affect the expression of ASPM201(Fig. 6. A & B). EdU staining demonstrated that the knockdown of ASPM203 inhibited the proliferation of the MUT group (Fig. 6. C). Colony formation and CCK-8 assay also showed that the MUT group regained tumor suppressor ability by downregulating ASPM203 (Fig. 6. D & E). The same effect was observed in wound healing and transwell assay (Fig. 6F & G). These results suggested that RBM10^C761Y^ promoted CCA progression depending on ASPM203.

### ASPM203 interacted with DVL2 to enhance Wnt/β-catenin signaling

**Fig. 7:**
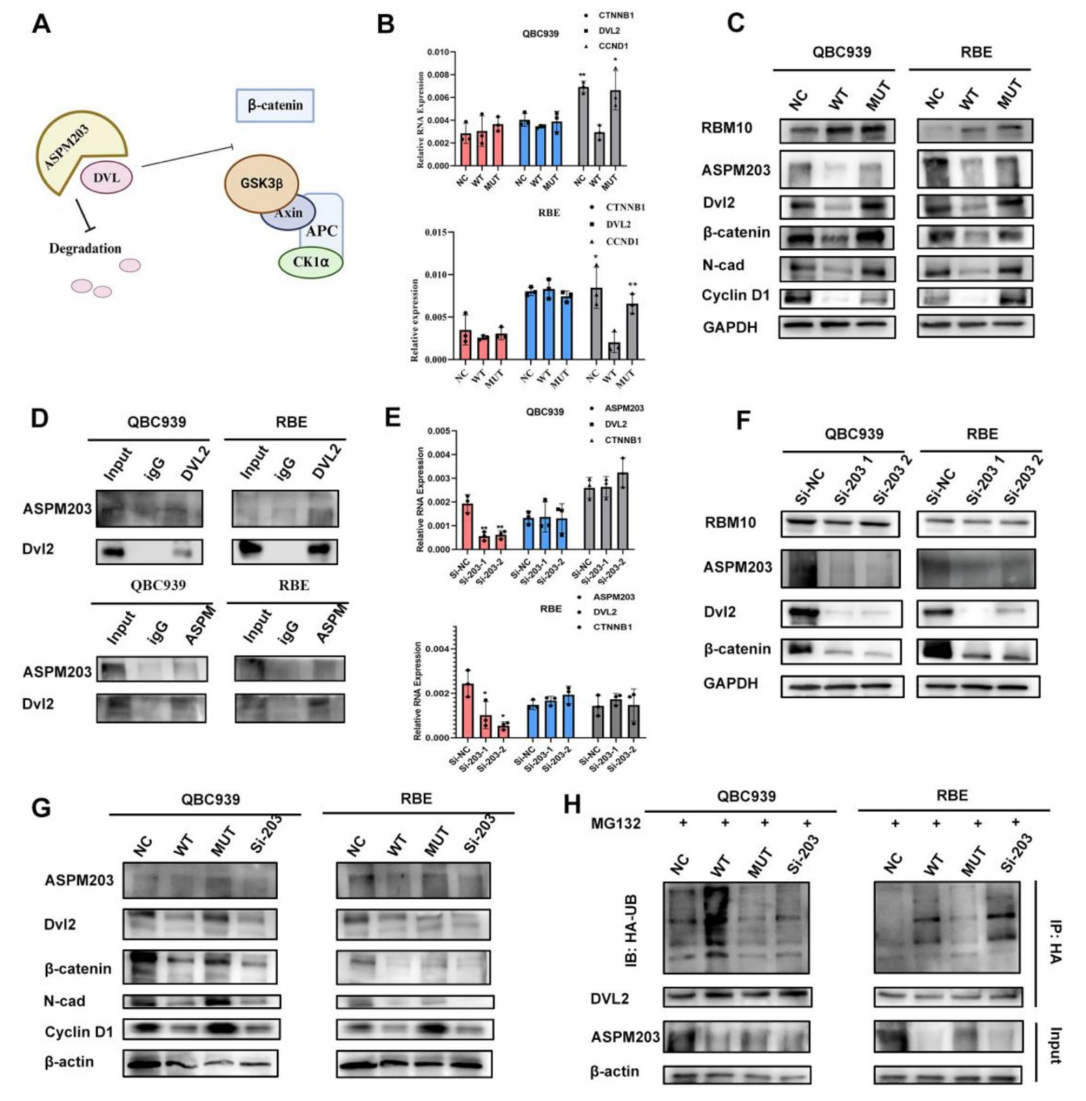
ASPM203 interacted with DVL2 to facilitate Wnt/β-catenin signaling. **A** The diagram illustrates the role of ASPM in regulating the Wnt signaling pathway, as reported in previous studies. **B** Co-immunoprecipitation was performed to verify the physical interaction between ASPM203 and dishevelled-2 (DVL2), a key mediator of Wnt/β-catenin signaling. **C**, **D** Using RT-PCR and western blot to determine the different levels of Wnt/β-catenin signaling associated genes in the NC, WT, and MUT groups. **E, F** Using RT-PCR and western blot to determine the different levels of Wnt/β-catenin signaling associated genes with knocking down of ASPM203 in the NC group. **G, H** Expression and ubiquitylation of DVL2 were detected in four groups, including the NC, WT, MUT, and ASPM203-knockdown groups

To explore the regulatory mechanism of ASPM203 in CCA, we analyzed the transcriptome sequencing data of the WT and MUT groups. GO term analysis showed that transcription regulation was also upregulated in the MUT group (Fig S4. F). Pathway enrichment analysis revealed that the Wnt/β-catenin signaling pathway was significantly upregulated in the MUT group compared with the WT group (Fig S4. G). Previous studies have shown that ASPM203 interacted with dishevelled (DVL) protein to participate in cancerogenesis [25, 26], which suggested that ASPM203 bound to DVL protein, preventing degradation and thus enhancing the Wnt/β-catenin signaling pathway (Fig. 7. A). Notably, co-immunoprecipitation assay revealed that ASPM was associated with DVL2 but not with DVL3 in CCA cells (Fig. 7. D & S3. E). We analyzed the changes of Wnt-related genes in NC, WT, and MUT groups and found that CTNNB1 (β-catenin) and DVL2 did not change at the mRNA level, while CCND1 (cyclin D1) decreased at the transcription level in the WT, and MUT group had no difference with NC group (Fig. 7. B). At the protein level, DVL2, β-catenin, cyclin D1, and N-cadherin all decreased in the WT group, while the MUT group had no difference with the NC group (Fig. 7. C). When knocking down ASPM203 in the NC group, DVL2 and CTNNB1 did not change significantly at the transcription level, but decreased significantly at the protein level (Fig. 7. E & F). Further, knocking down ASPM203 in the MUT group resulted in similar changes to the WT group at protein level(Fig. 7. G). Immunofluorescence experiments also showed that nuclear β-catenin was reduced in the WT group compared to the NC group, while there was no significant change in the MUT group, but knockdown of ASPM203 in the MUT group reduced the nucleus translocation of β-catenin (Fig S5. A). The main form of DVL2 protein degradation is the ubiquitin-proteasome pathway [27]. Subsequently, we transfected HA-tagged ubiquitin into cells that were treated with MG132 to inhibit DVL2 degradation. When immunoprecipitated with DVL2 antibody, the level of ubiquitinated DVL2 was increased in the WT group, while there was no difference between the MUT group and the NC group. Conversely, the knockdown of aspm203 in the MUT group resulted in the loss of the ability to protect DVL2 from degradation (Fig. 7. H). These results suggested that ASPM203 bound to DVL2 and reduced its degradation, thereby enhancing the Wnt signaling pathway.

### RBM10^C761Y^ -modulated ASPM203 promoted CCA progression in a Wnt/β-catenin signaling dependent manner

**Fig. 8:**
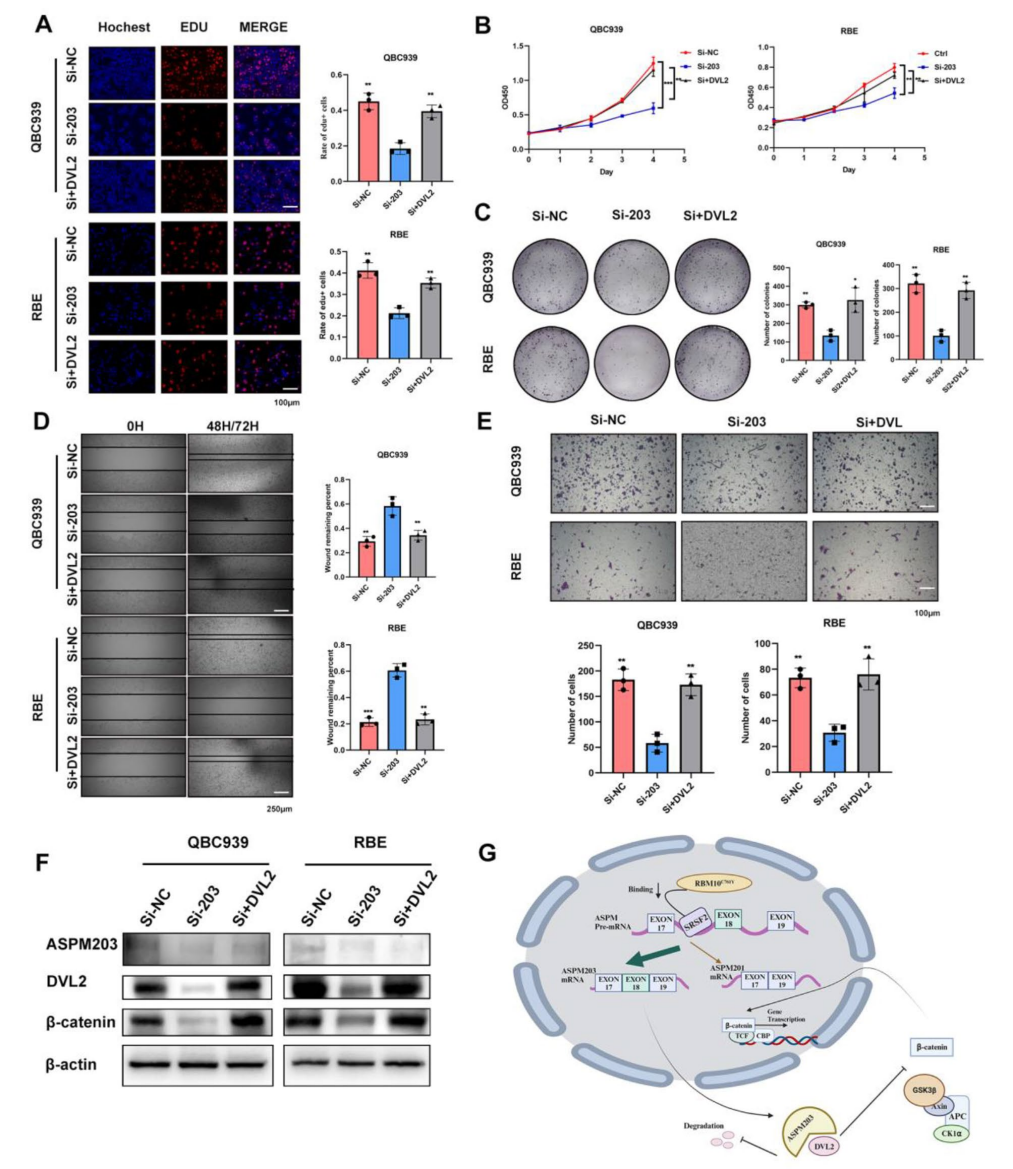
ASPM203 promoted CCA progression in a Wnt/β-catenin signaling dependent manner. **A, B, C** The alterations of CCA cell lines proliferation were evaluated by EdU, CCK-8, and colony formation assay in MUT, ASPM203-knockdown, and ASPM203-knockdown with DVL2-overexpression groups. **D, E** The alterations of CCA cell line migration were assessed by wound healing and transwell assay in MUT, ASPM203-knockdown, and ASPM203-knockdown with DVL2-overexpression groups. **F** The efficiency of ASPM203 knockdown and DVL2 overexpression were evaluated by western blotting. **G** Schematic representation of a model that RBM10 C761Y mutation induced oncogenic ASPM isoforms and regulated Wnt/β-catenin signaling in CCA

To explore how RBM10^C761Y^ modulated ASPM203 and enhanced CCA development in a β-catenin signaling dependent manner, we investigated the effect of ASPM203 silencing and DVL2 upregulation on CCA cell proliferation in two cell lines. In the MUT group, ASPM203 silencing significantly reduced cell proliferation, while in the DVL2 upregulation restored it, as shown by the EdU, CCk8, and colony formation assay (Fig. 8. A, B & C). In addition, we examined the effect of ASPM203 silencing and DVL2 upregulation on CCA cell migration in the MUT group: ASPM203 silencing abolished it, while DVL2 upregulation induced it, as evidenced by the wound healing and transwell assay (Fig. 8. D & E). The effect of DVL2 overexpression on β-catenin protein level was investigated in the ASPM203-silenced group: DVL2 overexpression could recover β-catenin protein level, as shown by the Western blot analysis (Fig. 8. F). The above results indicated that the C761Y mutation reduced the binding ability of RBM10 and SRSF2, leading to a decrease in ASPM exon18 ES event, an increase in ASPM203 expression, an enhancement of DVL2 stability, an upregulation of Wnt pathway, and a promotion of cholangiocarcinoma progression (Fig. 8. G).

## Discussion

In this study, we investigated the mutation spectrum of RBM10 in CCA. Using our previous and some domestic and international CCA genomic datasets, we found that RBM10 had a higher mutation rate in CCA among Chinese populations compared to Western populations. This was further validated in a recent study of the genomic profile of biliary tract tumors in the Chinese population (RBM10 mutation rate: 5.5%, 44/803) [28]. We originally intended to explore the etiological factors that make RBM10 more prone to mutations in Chinese population cohorts. However, the currently available public cohorts lack sufficiently comprehensive patient background information. In two Western population CCA cohorts from GEO database (GSE132305 and GSE26566), there was no significant difference in the expression levels of RBM10 between CCA and adjacent non-cancerous tissues. The above results implied that the pathogenesis of CCA varies across populations with distinct genetic and environmental factors, and these dimensions should be taken into account in CCA studies.

We also discovered that RBM10 mutations frequently coexisted with other driver gene mutations in CCA, suggesting that the widespread transcriptome alterations triggered by RBM10 mutations might cooperate with these mutations in the advancement of CCA (KRAS, SMAD4, CTNNB1, TGFBR2, SF3B1). It has been reported in LUAD studies that RBM10 mutations co-occur with EGFR mutations, and affect the response of LUAD patients to EGFR inhibitors by influencing the alternative splicing of the apoptotic protein BCL-X [29]. These results indicate that clarifying the mechanisms involved in RBM10 mutations will assist in developing approaches for addressing these mutations.

The majority of RBM10 mutations in CCA result in protein truncation and functional domain loss, which is consistent with the RBM10 findings in LUAD [30]. This characteristic of loss-of-function mutations is also exhibited by most of the tumor suppressor gene mutations [31]. However, there are also many missense mutations in these tumor suppressor genes, which affect various functional domains throughout the gene and lead to alterations in function, either loss or gain. RBM10 wild-type protein is highly conserved among mammals and functions as a splicing regulator and a tumor suppressor in various cancers [13, 20, 32]. This protein comprises two zinc fingers, two RNA recognition motifs, bipartite nuclear localization signals, one glycine-rich region, and an Octamer Repeat domain. We detected a C761Y mutation in the C_2_H_2_-type zinc finger domain, also reported in colorectal cancer (C761W) and lung cancer (C761Y) by the COSMIC database, but the influence was largely unknown.

To the best of our knowledge, this is the first study to show that RBM10 acts as a tumor suppressor both in vivo and in vitro and correlates with favorable outcomes in patients with CCA. The C761Y mutation caused the loss of RBM10 tumor suppressor ability. We then found that the C761Y mutation caused widespread changes at the transcriptional level, and the ES event of ASPM exon 18 was one of the most significant events. ASPM was originally identified as a centrosome-associated protein that modulates neural development. Data gathered over recent years revealed its multifaceted functions in cancer cellular processes [33, 34]. ASPM exhibits two major splicing variants comprising ASPM203 and ASPM201. In contrast to the most extensive isoform ASPM203, the smaller ASPM201 variant omits some domains, such as the isoleucine and glutamine motif and the calponin-homology domain, leading to diverse roles in normal and cancerous cells [25, 26]. Our research validated the contribution of ASPM203 for RBM10^C761Y^ in CCA progression. Previous studies have shown that ASPM was involved in the regulation of the Wnt canonical pathway [26, 33, 35], and our data also indicate that the Wnt/β-catenin pathway was upregulated in the MUT group. Further studies reveal that the RBM10^C761Y^ mutation led to an increase in ASPM203 RNA and protein levels, and the elevated ASPM203 stabilized DVL2 protein, upregulated the Wnt/β-catenin pathway, and promoted CCA cell progression. In summary, our findings showed that RBM10^C761Y^-modulated ASPM203 promoted CCA progression in a Wnt/β-catenin signaling dependent manner.

To further elucidate how RBM10 and C761Y mutation mediated the ASPM exon18 ES event, we reviewed the role of RBM10 in alternative splicing. In the splicing reaction of target pre-mRNAs, RBM10 interacts with the splice sites of cassette exons (within branchpoint sequence-containing intronic region upstream of cassette exons) and modulates the 3’- and 5’-splice site recognition. This results in the preferential selection of the 5’ and 3’-splice sites that are distal to the cassette exons, leading to the skipping of the cassette exons [20, 21, 36]. Our minigene splicing reporter assay demonstrated that RBM10 was involved in the regulation of the ASPM EXON18 ES event (the C761Y mutation reduced the exon skipping event and generated more ASPM203). However, previous CLIP-Seq data indicated that RBM10 binds to ASPM at a position that is not near the branchpoint sequence of the intron (Fig S3. B). Meanwhile, RBM10 was not detected in ASPM minigene pull-downs. Therefore, the regulation of ASPM ES event requires the participation of other alternative splicing auxiliary factors. Serine and arginine rich splicing factor 2 (SRSF2) interacting with wild-type RBM10 was involved in the spliceosome and promoted the ASPM EXON18 ES event, while the mutant RBM10 lost its binding ability to SRSF2 and failed to participate in the ASPM EXON18 ES event. Previous SRSRF2 CLIP-seq data also suggested its involvement in the ASPM EXON18 ES event (Fig S3. C) [37]. We therefore concluded that RBM10 mediated the ASPM ES event through its interaction with SRSF2, whereas RBM10^C761Y^ impaired the binding affinity.

## Conclusion

Our study revealed a critical putative driver gene, RBM10^C761Y^ that was not identified in previous studies. Additionally, we elucidated the regulatory mechanisms that link splicing variants to CCA. RBM10^C761Y^ mutation induced oncogenic ASPM isoforms and regulated Wnt/β-catenin signaling in CCA, which pointed to possible therapeutic targets. However, several problems are interesting to investigate. The spliceosome, a highly complex, dynamic, and protein-rich ribonucleoprotein complex, catalyzes pre-mRNA splicing by assembling de novo on each intron. Various splicing factors participate in the ASPM exon18 ES event. The stringent conditions we applied in the pull-down assay eliminated many of them. Moreover, RBM10 modulates a wide range of gene alternative splicing events, which may affect the alternative splicing of other genes besides ASPM, leading to unforeseen consequences. This possibility should not be overlooked, and thus, additional research was warranted.

### Electronic supplementary material

Below is the link to the electronic supplementary material.

Supplementary Material 1

Supplementary Material 2

Supplementary Material 3

Supplementary Material 4

Supplementary Material 5

## Data Availability

The experimental data presented in the study are included in the article/ Supplementary Materials, further inquiries can be directed to the corresponding authors upon reasonable request.

## Abbreviations

ASPM: Assembly Factor For Spindle Microtubules
CCA: Cholangiocarcinoma
CLIP: Cross-Linking Immunoprecipitation
DVL: Dishevelled Protein
ES: Exon Skipping
LUAD: Lung Adenocarcinoma
MS: Mass Spectrometry
RBM10: RNA Binding Motif protein 10
SRSF2: Serine And Arginine Rich Splicing Factor 2
TCGA: The Cancer Genome Atlas
