# Supplementary material for: RBM10 C761Y mutation induced oncogenic ASPM isoforms and regulated β-catenin signaling in cholangiocarcinoma
Source: J Exp Clin Cancer Res. 2024 Apr 4;43:104. doi: 10.1186/s13046-024-03030-x (PMC10993532; doi:10.1186/s13046-024-03030-x)
Supplement: Supplementary file 4 — Supplementary Material 4 [file 13046_2024_3030_MOESM4_ESM.docx]

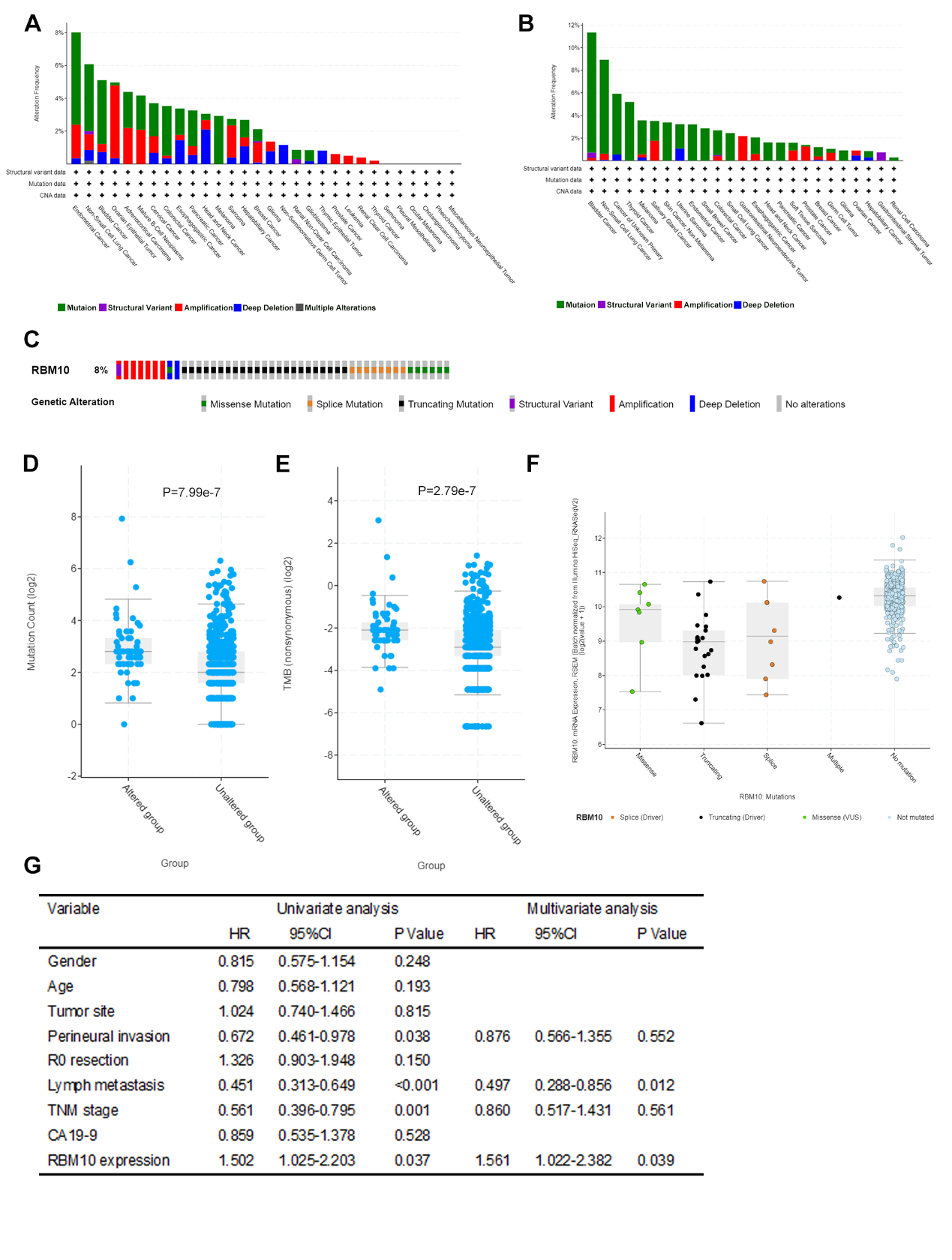


**Figure S1. A** RBM10 alteration frequency in pan-cancer in TCGA cohort. **B** RBM10 alteration frequency in pan-cancer in MSK-IMPACT cohort. **C** The mutation pattern of RBM10 in the TCGA lung adenocarcinoma population. **D, E** The different mutation count and tumor mutation burden between alter and unalter groups in the Chinese CCA population. **F** RBM10 mRNA expression in TCGA lung adenocarcinoma samples with different types of mutations, without mutation. **G** Univariate and multivariate analyses of prognostic factors in TMA CCA patients.


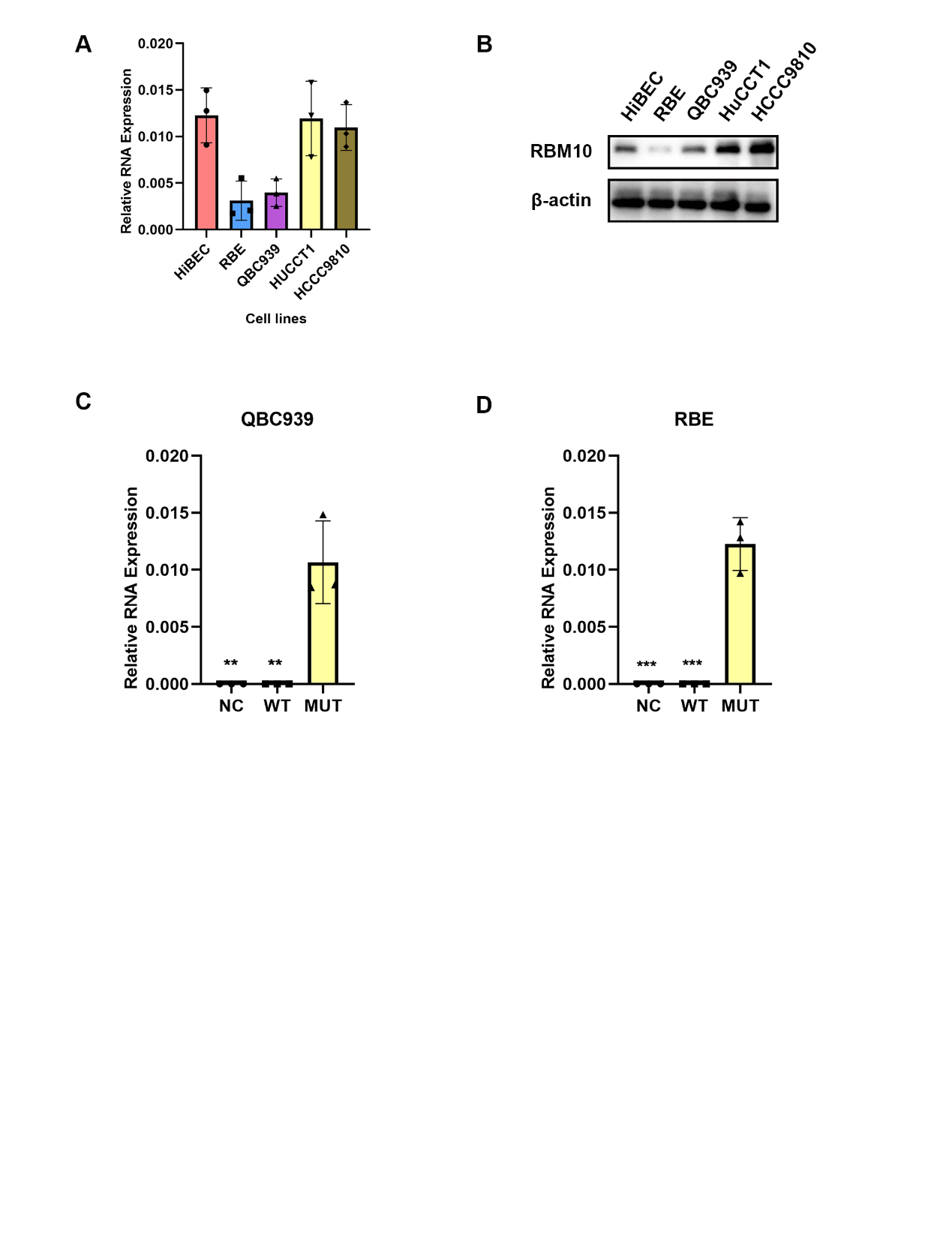


**Figure S2. A, B** The mRNA and protein expression levels of RBM10 in four CCA cell lines (QBC939, RBE, HuCCT1, and HCCC9810) and one normal bile duct cell line (HiBEC). **C, D** The mRNA expression levels of RBM10 C761Y in QBC939 and RBE.


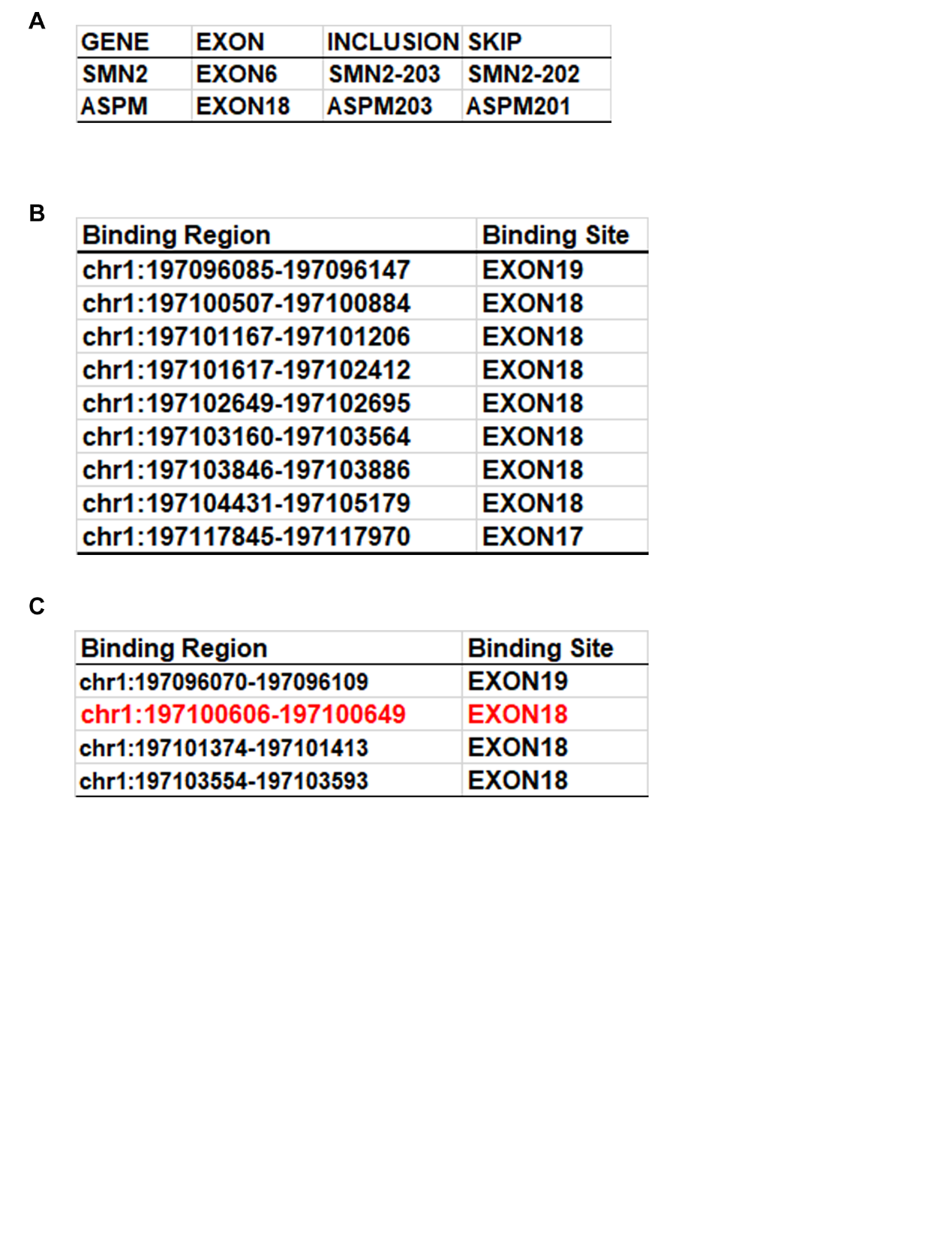


**Figure S3. A** Two potential AS targets of RBM10 C761Y mutation in CCA cells. **B, C** CLIP-seq data predict the binding site of RBM10 and SRSF2 on ASPM-pre-mRNA from the ENCORI database.


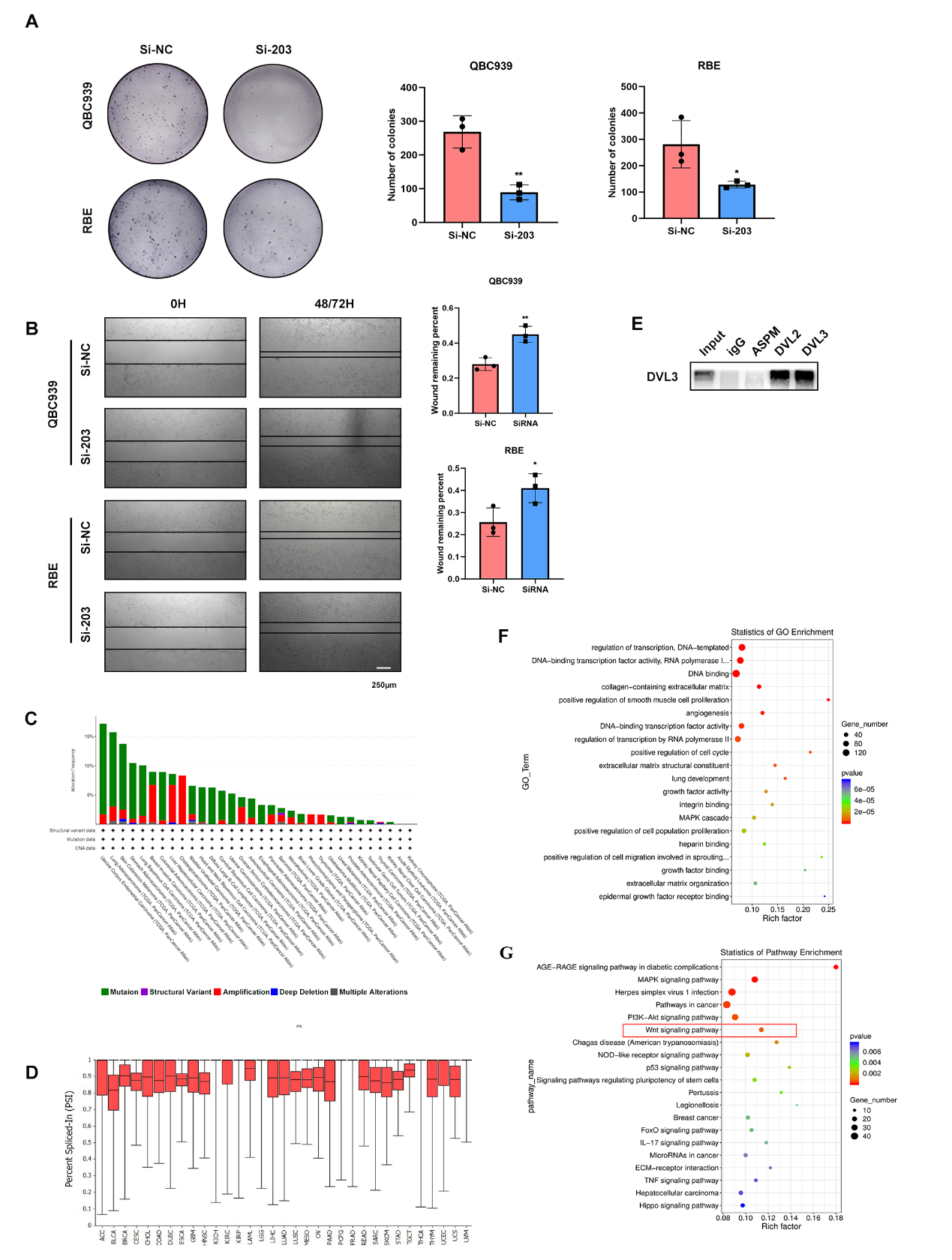


**Figure S4. A, B** The evaluation of the role of ASPM203 in CCA progression with ASPM203 knocking down in NC group by colony formation and transwell assay. after potential AS targets of RBM10 C761Y mutation in CCA cells. **C** ASPM alteration frequency in pan-cancer in TCGA cohort. **D** ASPM PSI data in TCGA pan-cancer cohort. **E** Co-immunoprecipitation was performed to verify the physical interaction between ASPM203 and DVL3. **F, G** GO term analysis and KEGG pathway of RNA-seq data from the WT and MUT groups showed the top 20 enriched biological processes.


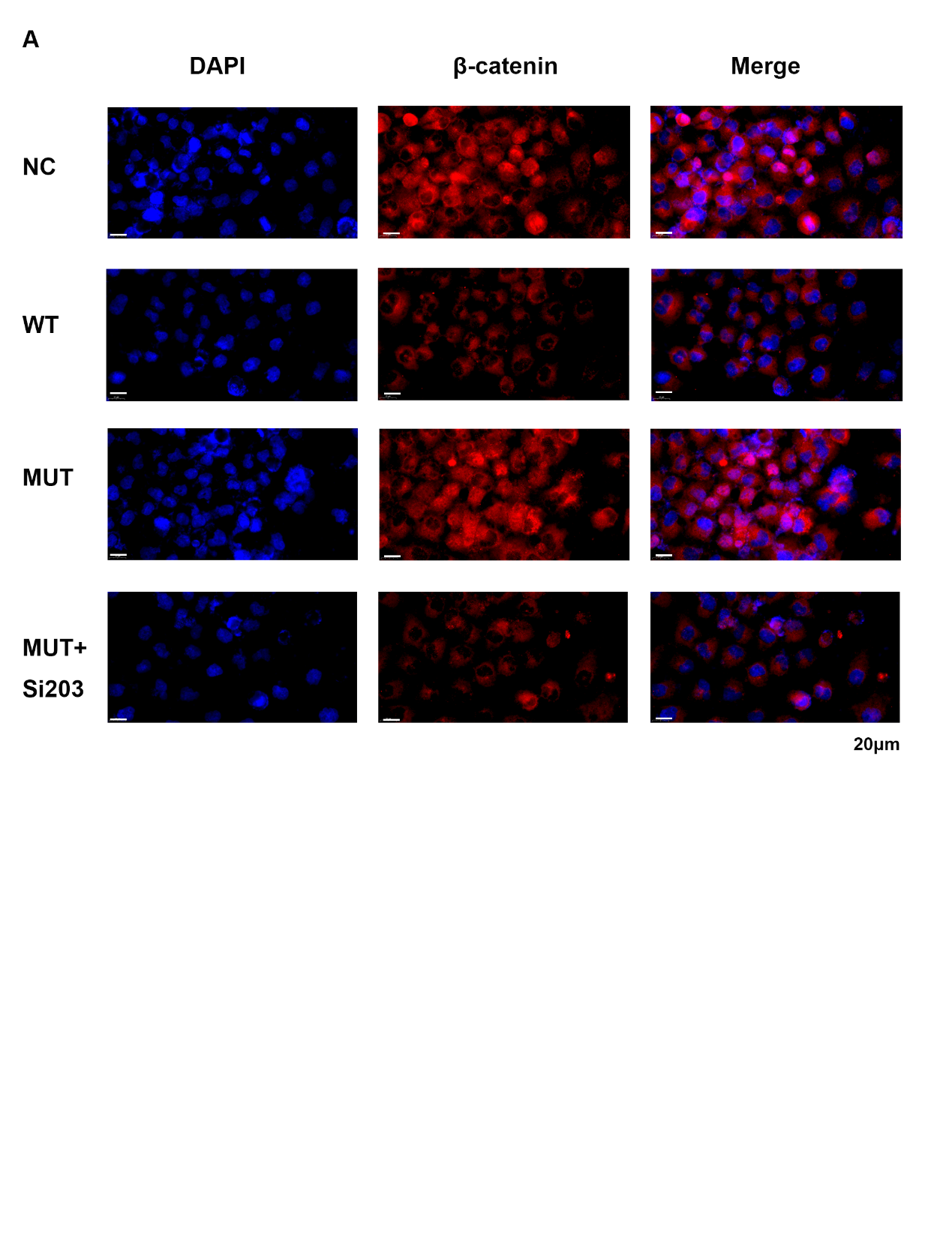


**Figure S5. A** Representative immunofluorescence images of β-catenin in the NC, WT, MUT, and ASPM knocking-down Group in QBC939.
