# Supplementary material for: RBM10 C761Y mutation induced oncogenic ASPM isoforms and regulated β-catenin signaling in cholangiocarcinoma
Source: J Exp Clin Cancer Res. 2024 Apr 4;43:104. doi: 10.1186/s13046-024-03030-x (PMC10993532; doi:10.1186/s13046-024-03030-x)
Supplement: Supplementary file 5 — Supplementary Material 5 [file 13046_2024_3030_MOESM5_ESM.docx]

March 17th, 2024

Dear editorial board of Journal of Experimental & Clinical Cancer Research:

On behalf of my co-authors, we appreciate your and the reviewers’ invaluable comments concerning our manuscript entitled “RBM10 C761Y mutation induced oncogenic ASPM isoforms and regulated β-catenin signaling in cholangiocarcinoma” (JECC-D-24-00247). These comments are all constructive and valuable for improving our manuscript. The supplementary experiments we conducted were sponsored by the Natural Science Foundation of Jiangsu Province (BK20220723). We have studied comments carefully and have made revisions which highlighted in track changes of Word in the manuscripts.

**Response to Reviewer #1:**

The manuscript by Chang et al. is a well-designed study focused on understanding the role of RBM10 and its mutations (more specifically the mutation C761Y) in cholangiocarcinoma (CCA) pathobiology. By applying multiple cell and molecular biology techniques and in vivo experimental approaches, they described the role of RBM10-C761Y in regulating the alternative splicing of ASPM, which in turn stabilizes DVL2 to enhance beta-catenin signaling and, thus CCA growth. Although in general the manuscript is impeccable and easy to follow, there is a few aspects that should be considered before it is ready for publication in JECCR.

**[Response]** Thank you for your interest in our research. For your constructive suggestions regarding our research, we will give detailed revisions in the answers below.

**[Comment 1]** The authors indicate that RBM10 mutations occur more frequently in Chinese patients. Is this related to CCA etiology (i.e. liver fluke) in those regions compared with western countries?

**[Response]** Thank you for your helpful comment. In 2017, a study proposed the molecular characteristics of CCA and its close association with tumor etiology^[1].^ This study primarily elucidated the genomic features of CCA related to liver fluke infection. However, the research employed targeted sequencing of 404 genes, which did not include RBM10. Thailand is an area with a high prevalence of liver fluke infection. However, a targeted sequencing study on Thai individuals with bile duct cancer still did not include RBM10^[2]^.The datasets from China pan-cancer cohort and MSK-IMPACT Clinical Sequencing Cohort do not include enough environmental carcinogens to analyze the reasons behind differential mutational features of RBM10 across distinct populations. Therefore, we conducted some analyses using another cohort of Chinese population, as mentioned in the Discussion section of the article^[3]^. In this cohort, the mutation rate of RBM10 in bile duct cancer is 6.3% (40/639). The cohort had information about HBV/HCV infection in some of the patients, which we analyzed in relation to the RBM10 mutation and did not find a significant correlation (P>0.05). The currently available public cohorts lack sufficiently comprehensive patient background information. Therefore, the potential etiological reasons for the elevated RBM10 mutation frequency in certain Chinese populations need further exploration in future studies. We have added these in the Discussion section **(line 413-416)**.

| The relationship between HBV/HCV infection and RBM10 mutation | | | | | |
| --- | --- | --- | --- | --- | --- |
|  |  | RBM10 Mutation | |  |  |
|  |  | Positive | Negative | Total |  |
| HBV/HCV  infection | Positive | 7 | 192 | 199 |  |
|  | Negative | 30 | 373 | 403 | P>0.05 |
|  | Total | 37 | 565 | 602 |  |

**[Comment 2]** The authors show a decrease in the expression of RBM10 in CCA tumors compared to non-tumoral surrounding tissue. Does this happen more frequently in Chinese patients compared to western countries? Or, different from the mutations, is a generalized phenomenon.

**[Response]** Thank you for your kind advice. We conducted analyses using two western population datasets from GEO (GSE132305 and GSE26566). In the two cohorts, there was no significant difference in the expression levels of RBM10 between CCA and adjacent non-cancerous tissues (p>0.05). The validation of this result requires a larger-scale cohort. This may reveal that RBM10 plays a more important role in some Chinese population, which will be a good question to be explored in our future study. We have added these in the Discussion section **(line 416-419).**

**[Comment 3]** Following the previous question. The authors show that RBM10 expression is downregulated in tumor CCA tissues compared to non-tumoral surrounding tissue. Then, they start to analyze the role of RBM10 in CCA by increasing its expression in CCA cell lines. It would be interesting to validate that CCA cell lines used in this study express less RBM10 than normal cholangiocytes.

**[Response]** To mitigate the influence of background RBM10, we assessed the expression levels of RBM10 in four CCA cell lines (QBC939, RBE, HuCCT1, and HCCC9810) and one normal bile duct cell line (HiBEC) before establishing experimental cell models. We selected two cell lines, QBC939 and RBE, which exhibited the lowest expression levels of RBM10. We have added these in **Figure S2(line 255-259).**

**Figure S2. A, B** The mRNA and protein expression levels of RBM10 in four CCA cell lines (QBC939, RBE, HuCCT1, and HCCC9810) and one normal bile duct cell line (HiBEC).

**[Comment 4]** In line 74 the word "identified" is missing between "also" and "several".

**[Response]** Thank you for your keen comments. We have revised these mistakes in our revised manuscript.

**[Comment 5]** In line 434 there is an extra space between paragraphs.

**[Response]** Thank the reviewer for pointing this out. We have revised this mistake in the revised manuscript.

**[Comment 6]** In Figure 4C the word "exclueded" must be corrected.

**[Response]** We have to say sorry about the troubles brought by our typos. We have corrected it in our revised manuscript.

[1] Jusakul A, Cutcutache I, Yong CH, et al. Whole-Genome and Epigenomic Landscapes of Etiologically Distinct Subtypes of Cholangiocarcinoma[J]. Cancer Discov 2017,7(10):1116-1135.

[2] Chaisaingmongkol J, Budhu A, Dang H, et al. Common Molecular Subtypes Among Asian Hepatocellular Carcinoma and Cholangiocarcinoma[J]. Cancer Cell 2017,32(1):57-70 e3.

[3] Lin J, Cao Y, Yang X, et al. Mutational spectrum and precision oncology for biliary tract carcinoma[J]. Theranostics 2021,11(10):4585-4598.

**Response to Reviewer #2:**

It's my pleasure to review this manuscript by Chang et al. In this manuscript, the authors demonstrated that C761Y mutation inhibited the proper function of RBM10. The ratio of ASPM transcripts was regulated by RBM10-SRSF2 complex. Finally, irregulated transcripts lead to abnormal activation of Wnt pathway by interaction with DVL2. Overall, the conclusion was reliable, because many techniques were applied. However, the regulatory axis was long and complex. Therefore, some revisions should be made.

**[Response]** We would like to express our sincere appreciation for your careful reading and valuable comments to improve our manuscript.

**[Comment 1]** This manuscript mainly focused on the functional mutation of RBM10. However, it seems that only wild-type RBM10 (or total RBM10?) was examined in tissue samples in figure 2. Direct evidences are required for the expression of mutated RBM10 by newly developed antibodies or other methods, e.g., qPCR.

**[Response]** Thank you for your suggestion. In the preliminary research, we indeed used designed primers and Sanger sequencing to detect the presence of the C761Y mutation in 36 CCA samples which were used in tissue microarrays. However, we did not identify any RBM10^C761Y^ mutations among them. RBM10 mutations are dispersed throughout various regions of the gene. To determine their presence in the samples using Sanger sequencing, we would need to design at least 24 pairs of primers corresponding to the relevant exons, which is resource-intensive. Consequently, in our subsequent studies, we aimed to perform transcriptome sequencing on CCA samples. For certain functionally significant RBM10 mutations including C761Y, we will design specific antibodies targeting them. These approaches allow us to explore the genetic stability of these RBM10 mutations and their impact on prognosis.

**[Comment 2]** The authors demonstrated that both CCA cell lines carried RBM10 mutations. Why was RBM10 found to immunoprecipitate ASPM203 in both cell lines using specific antibodies as the RBM10 protein was mutated? The result of pull-down assay might be indicative for this problem (Line 318).

**[Response]** We apologize for any confusion caused by our previous description. The immunogen recognized by the antibody used in our research (Novus, NB100-55265) maps to a region between residue 880 and the C-terminus (residue 930) of RBM10. Whether in the two-dimensional structure or the three-dimensional structure predicted by AlphaFold, the C761Y mutation is indeed distant from the immunogen area. Therefore, the C761Y mutation may not have an impact on the antibody. In the RNA immunoprecipitation (RIP) experiment, we assessed whether RBM10 binds to the entire ASPM mRNA sequence. In the pull-down assay, we used an ASPM minigene (comprising only truncated exons 17-19 and flanking introns). The lack of direct binding between RBM10 and these sequences likely influenced the Western blot results. We have admitted that lacking cross-linking immunoprecipitation (CLIP) technology to precisely determine the specific binding sites of RBM10 in regulating RNA is a limitation of this study. We have added these in the Methods section **(line 170-172).**

**[Comment 3]** Figure 3: The post-transcriptional view of this manuscript is interesting. However, as we know, the alternative splicing process was complex and the product could not be predicted especially when long transcripts were ectopically expressed. I think it necessary to make sure the products if mutated RBM10 transcripts were overexpressed to exclude the additional splicing, which is particularly essential for transcript study.

**[Response]** Thank you for your helpful comment. In the transcriptome sequencing data of the MUT group, we detected mutations in RBM10 within the MUT groups. Additionally, we designed mutation-specific primers to assess the expression levels of mutated transcripts. The C761Y mutation was detected exclusively in the MUT group. (Forward primer:GGACATAAGGAGACAGGGGAA; Reverse primer: CTTGGCAATCTGTTGAGCTGTC). We have added these in **Figure S2 (line 263-264).**

| **RNA-seq results in the NC, WT, MUT Group** | | | | | | | | | | | | |
| --- | --- | --- | --- | --- | --- | --- | --- | --- | --- | --- | --- | --- |
| Chr | Position | Ref | Transcript | mut1 | mut2 | mut3 | nc1 | nc2 | nc3 | wt1 | wt2 | wt3 |
| X | 47185557 | G | ENST00000377604 | G->A | G->A | G->A | - | - | - | - | - | - |

**Figure S2 C, D** The mRNA expression levels of RBM10 C761Y in QBC939 and RBE.

**[Comment 4]** The expression level of β-Catenin is not the essential condition for Wnt pathway activation. Maybe other parameters could be considered, e.g, the expression level of target genes or β-Catenin transport.

**[Response]** We thank the reviewer for this insightful suggestion. We assessed the nuclear localization of β-catenin in the NC, WT, and MUT groups through immunofluorescence experiments. Additionally, we examined the nuclear localization of β-catenin after knocking down ASPM203 in the MUT group. The assay showed that nuclear β-catenin was reduced in the WT group compared to the NC group, while there was no significant change in the MUT group, but the knockdown of ASPM203 in the MUT group reduced the nucleus translocation of β-catenin. We have added these in **Figure S5 (line 378-381).**

**Figure S5. A** Representative immunofluorescence images of β-catenin in the NC, WT, MUT, and ASPM knocking-down Group in QBC939.

**[Comment 5]** The sheet of antibody could be moved into the supplemental materials.

**[Response]** Thanks for your suggestion. We have moved the sheet of antibody into supplemental materials.

**[Comment 6]** In line 163, the citation for figure is inappropriate in method section.

**[Response]** Thanks for your advice. We have moved it to **Table S3**.

**[Comment 7]** Line 299-302. The description is confusing.

**[Response]** We apologize for any confusion caused by our previous description. We have rephrased this statement. In both the WT and MUT groups, we employed RNA immunoprecipitation experiments to determine which gene specifically binds to RBM10. The experimental results revealed that ASPM interacts with both the wild-type RBM10 and the C761Y mutant, however, the binding capacity of the C761Y mutant to ASPM was diminished. These results implied that RBM10 may be involved in the ASPM ES event and C761Y mutant impaired the interaction between ASPM and RBM10 **(line 304-310).**

**[Comment 8]** Many spelling mistakes were found, e.g., "proteun" in Figure 2C, "exclueded" in Figure 4C, "Edu" in Line 344, and "Protein" in Line 256.

**[Response]** Thank you very much for your comments on the details of our article. We carefully reviewed the article for spelling errors and made the necessary corrections.

We would like to express our great appreciation for you and the reviewers’ warm work earnestly. In particular, we would like to express our sincere thanks to you for your understanding and kindness during our submission.

Best regards

Xiangcheng Li, M.D., Ph.D.

Professor of Hepatobiliary Surgery

Hepatobiliary Center, The First Affiliated Hospital of Nanjing Medical University

300 Guangzhou Road, Nanjing 210029, China

Tel (Fax): 86-25-6830-3226

Email: drxcli@njmu.edu.cn
